# Assessing adequacy of citizen science datasets for biodiversity monitoring

**DOI:** 10.1002/ece3.10857

**Published:** 2024-01-31

**Authors:** Louis J. Backstrom, Corey T. Callaghan, Nicholas P. Leseberg, Chris Sanderson, Richard A. Fuller, James E. M. Watson

**Affiliations:** ^1^ School of the Environment, Centre for Biodiversity and Conservation Science The University of Queensland St Lucia Queensland Australia; ^2^ School of Mathematics and Statistics, Centre for Research into Ecological and Environmental Modelling The University of St Andrews St Andrews UK; ^3^ Department of Wildlife Ecology and Conservation, Fort Lauderdale Research and Education Center University of Florida Davie Florida USA; ^4^ Research and Recovery of Endangered Species Group The University of Queensland St Lucia Queensland Australia

**Keywords:** adequacy, citizen science, community science, data bias, eBird, inventory completeness

## Abstract

Tracking the state of biodiversity over time is critical to successful conservation, but conventional monitoring schemes tend to be insufficient to adequately quantify how species' abundances and distributions are changing. One solution to this issue is to leverage data generated by citizen scientists, who collect vast quantities of data at temporal and spatial scales that cannot be matched by most traditional monitoring methods. However, the quality of citizen science data can vary greatly. In this paper, we develop three metrics (inventory completeness, range completeness, spatial bias) to assess the adequacy of spatial observation data. We explore the adequacy of citizen science data at the species level for Australia's terrestrial native birds and then model these metrics against a suite of seven species traits (threat status, taxonomic uniqueness, body mass, average count, range size, species density, and human population density) to identify predictors of data adequacy. We find that citizen science data adequacy for Australian birds is increasing across two of our metrics (inventory completeness and range completeness), but not spatial bias, which has worsened over time. Relationships between the three metrics and seven traits we modelled were variable, with only two traits having consistently significant relationships across the three metrics. Our results suggest that although citizen science data adequacy has generally increased over time, there are still gaps in the spatial adequacy of citizen science for monitoring many Australian birds. Despite these gaps, citizen science can play an important role in biodiversity monitoring by providing valuable baseline data that may be supplemented by information collected through other methods. We believe the metrics presented here constitute an easily applied approach to assessing the utility of citizen science datasets for biodiversity analyses, allowing researchers to identify and prioritise regions or species with lower data adequacy that will benefit most from targeted monitoring efforts.

## Abstract 2

Citizen science data are increasingly being used to monitor biodiversity, but datasets produced by citizen scientists come with a number of well‐recognised challenges. In this paper, we develop several metrics to assess the adequacy of spatial observation data from citizen science projects and explore these metrics at the species level for Australia's terrestrial birds. We find that data adequacy for most Australian birds is increasing, but several gaps still remain in the spatial coverage of citizen science data across the Australian continent.

## 1 INTRODUCTION

Biodiversity monitoring is an essential component of conservation (Lindenmayer et al., 2012). Data collection is the foundation of successful monitoring, and when effectively collected and analysed, occurrence data can inform the status and trend of species, allowing conservation practitioners and policy makers to identify taxa most in need of targeted management interventions. However, resources available to formal monitoring schemes are typically very limited (Kuebbing et al., 2018), meaning that the majority of species are not well monitored across their entire ranges, and in many cases, the limits of their range may not be fully known (Whittaker et al., 2005). Even for relatively well‐studied taxa such as birds, or regions such as North America, Europe, or Australia, these issues are pervasive (Backstrom et al., 2023). In light of this, a key challenge facing biodiversity and conservation science is prioritising species and regions in need of additional monitoring resources (Wilson et al., 2015).

One increasingly popular approach to biodiversity monitoring is to leverage data generated by volunteers through citizen science programmes. Citizen science has led to numerous advances in understanding species' populations in space and time (e.g. Johnston et al., 2020; Van Strien et al., 2013), and many visions for biodiversity monitoring that take advantage of citizen science have been promoted at local, regional and global scales (Chandler et al., 2017; Pocock et al., 2018). Despite this promise, there are several obstacles limiting the widespread adoption of citizen science (Burgess et al., 2017), including real or perceived data quality issues leading to a lack of trust by analysts and policymakers (Binley & Bennett, 2023), and statistical challenges during the analysis of citizen science data (Johnston et al., 2023). In most circumstances, overcoming these issues will require some degree of data integration—that is, using broad‐scale citizen science data in combination with localised high‐quality data collected by specialists. To achieve this integration, understanding the adequacy of citizen science datasets in this context is an important first step.

In this paper, we provide methods to assess data adequacy in two different contexts: completeness (two metrics) and bias. For the most part, these two contexts have been well‐studied across various citizen science datasets (Deacon et al., 2023; Kelling et al., 2019; La Sorte & Somveille, 2020; Shirey et al., 2021), but to date, few studies have quantified data adequacy at a per‐species level—instead tending to explore them across spatial and temporal scales, identifying, for example, poorly sampled regions or temporal periods (Callaghan et al., 2022; Girardello et al., 2019), or exploring adequacy at higher taxonomic levels (Di Cecco et al., 2021; Mesaglio et al., 2023). We extend this body of literature by quantifying adequacy at the species level for Australia's terrestrial native birds. The advantage of the species‐level approach we present here is that adequacy can be directly related to the monitoring and conservation of individual species, allowing for frameworks which can (1) identify species for which currently available data may be ‘ready to go’ and can be used as‐is for modelling and tracking of populations and (2) prioritise species where investment in a ‘boots on the ground’ approach may be needed to fill knowledge gaps and establish a more complete picture of that species' conservation status (Backstrom et al., 2023).

We provide three metrics that can be used to assess data adequacy at a per‐species level in citizen science datasets. We then use data from two major Australian citizen science projects to calculate these metrics for all native Australian land birds. Considering the results of these analyses, we assess the value of each metric in the context of current knowledge on the distribution, abundance and status of Australia's birds and how each of the two Australian citizen science projects compare. We then model these metrics against a suite of seven species traits to identify any patterns and predictors of data adequacy that may permit a better understanding of data adequacy. Finally, we identify species and traits for which these analyses produced unexpected results, consider the possible reasons for these results and discuss whether these metrics could provide important novel information that improves our ability to assess the distribution, abundance and conservation status of species.

## 2 MATERIALS AND METHODS

### 2.1 Datasets

We used species occurrence data from two major citizen science programmes, eBird (Cornell Lab of Ornithology, 2022) and Birdata (Birdlife Australia, 2023). These programmes are the two largest (by volume) ecological citizen science datasets in Australia and collectively make up *c*. 75% of all bird records in the Atlas of Living Australia (https://www.ala.org.au). The two platforms are similar in that they are both semi‐structured (Kelling et al., 2019), allowing users flexibility in when, where and how they survey birds. However, Birdata tends to offer slightly more direction to users, in particular through a number of more structured protocols (e.g. 2‐ha, 20‐min surveys) and broad‐scale programmes with more specific data collection aims (e.g. Powerful Owl Project, Beach Nesting Birds Project, New Atlas of Australian Birds). In contrast, eBird typically offers very little specific direction to users beyond a small number of general suggestions (e.g. lists should be under 5 miles/8 km in length and 3 h in duration). The commonalities in structure between eBird and Birdata allow for data from the two platforms to be readily combined in analyses such as these, although analysts ought to be mindful of the differences between the two, as we show here.

**FIGURE 1:**
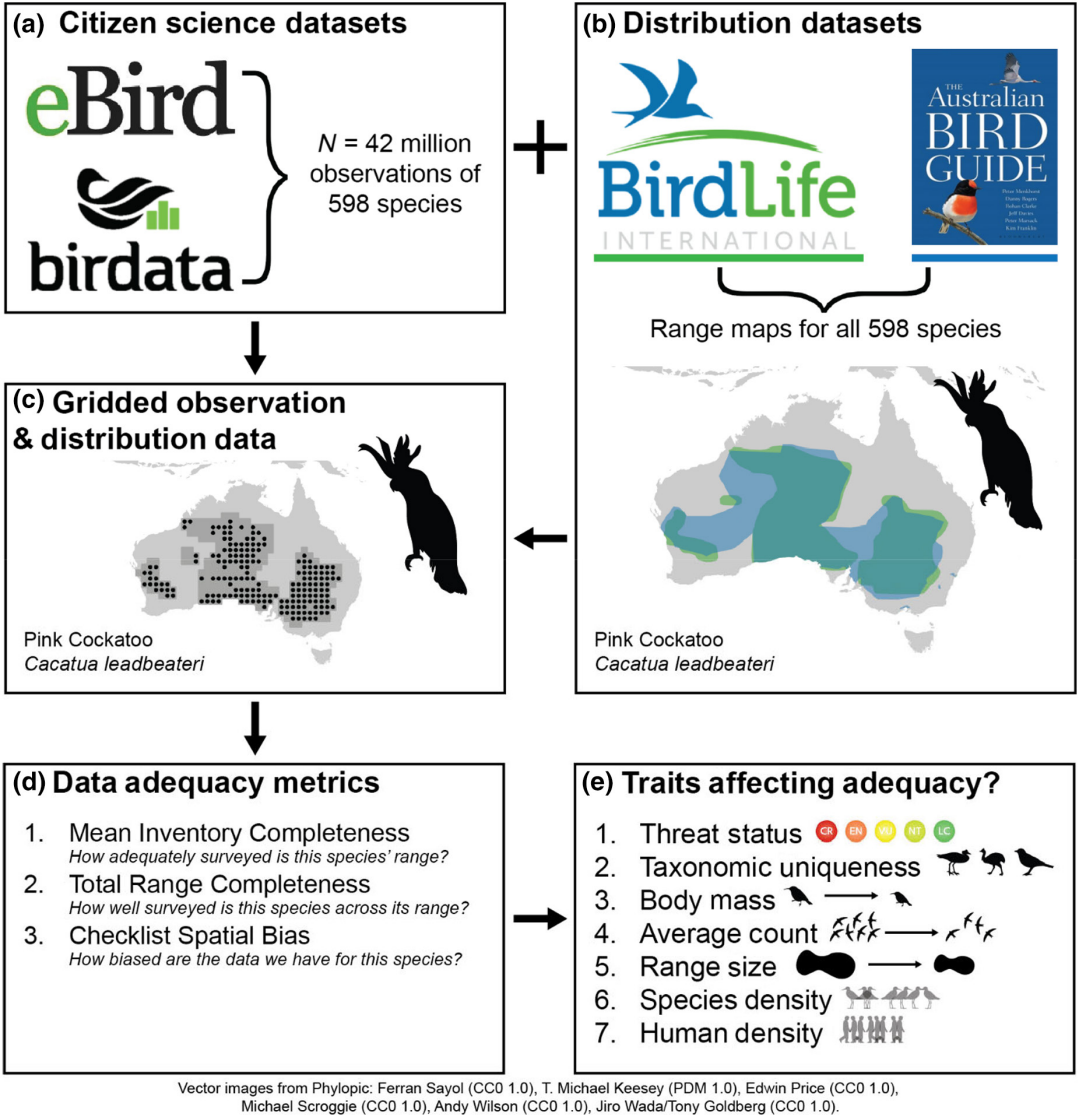
Methods framework used in this analysis. Occurrence data from two major citizen science programmes, and distribution data from two sources, were combined to produce three metrics of data adequacy. The relationships of seven species traits with each of these metrics were then modelled.

We filtered the two datasets to include only records from mainland Australia and offshore territories; marine species were excluded. Using the Working List of Australian Birds v2 (Birdlife Australia, 2017) as a reference, we excluded occurrences of species classified as exotic, vagrant or extinct, and filtered out data from non‐complete checklists (i.e. incidental observations). Duplicate copies of shared checklists (i.e. lists from people who were birdwatching together) were removed from the eBird dataset. We did not filter by other effort variables (e.g. duration, distance). We included records from all years (historical sightings were included), and we did not conduct any additional error checking beyond what is already done by the two programmes. We used species distribution data from two sources: Birdlife International (BirdLife International & Handbook of the Birds of the World, 2020) and the Australian Bird Guide (Menkhorst et al., 2017). These represent the most up‐to‐date spatial datasets of Australian bird distributions. Species distributions were combined across the two sources, clipped to mainland Australia and offshore territories, then filtered to only include extant, non‐vagrant ranges. Occurrences of species outside their mapped distributions were assumed to be vagrants and were removed from the combined occurrence dataset. We used the Working List of Australian Birds v2 (Birdlife Australia, 2017) as our baseline taxonomy, but as each of our four datasets uses a different taxonomic list, we had to manually resolve conflicting taxonomic arrangements, generally by combining ambiguous taxa at the lowest possible level. The combined occurrence dataset contained approximately 42 million observations referring to 598 species (Figure 1, panel a) and the combined species distribution maps covered all 598 species in the occurrence data (Figure 1, panel b). To facilitate the calculation of our data adequacy metrics, we simplified all datasets to a 1‐degree grid of the Australian region (Figure 1, panel c). This spatial scale was chosen as a balance between being fine enough to permit spatially relevant conservation and management conclusions, while being coarse enough to allow for sufficient data aggregation and for processing to complete in a manageable time frame. We tested the effect of changing this scale (at 2.0, 1.0, 0.5 and 0.2 degrees) and observed similar relative trends across metrics (Figures S1–S4).

### 2.2 Adequacy metrics

We present three adequacy metrics, aimed at answering different questions (Figure 1, panel d).

#### 2.2.1 Mean inventory completeness

Mean inventory completeness (MIC) answers the question ‘how adequately surveyed is this species' range’?. It is defined as the average (mean) inventory completeness of all grid cells across a species' range, where inventory completeness (in this instance) is the proportion of the *observed* diversity (the number of species recorded) relative to the *expected* diversity (the number of species mapped) in a given cell. Cells with sufficient sampling effort to detect the entire assemblage of species expected to be present will have high inventory completeness (values close to 1), and therefore species whose ranges overlap with these well‐surveyed cells will have high mean inventory completeness scores, suggestive of a species whose range has been adequately surveyed.

#### 2.2.2 Total range completeness

Total range completeness (TRC) answers the question ‘how well surveyed is this species across its range’?. It is defined as the proportion of a species' mapped range for which there are records of the species at the chosen grid cell grain (in our case, 1 degree). Species with high total range completeness (values close to 1) have records across much of their range, indicating that the species is well surveyed across its range, and likely allowing for more robust modelling of distribution and abundance.

#### 2.2.3 Checklist spatial bias

Checklist spatial bias (CSB) answers the question ‘how biased are the data we have for this species’?. It is defined as the spatial sampling bias of checklists in each cell across a species' range, following the methods developed by Backstrom (2022). Here, bias is the proportional difference between the observed distribution of sampling effort (in this instance, number of checklists in each grid cell across a species' range) and an expected (null) distribution (i.e. uniform spatial distribution of sampling effort across a species' range); more details are provided in Backstrom (2022). In this paper, we present spatial bias as the inverse of the bias of Backstrom (2022), that is 1−H, so as to keep the same directional coding as the other two measures (higher is better). As strong sampling bias can impede modelling (Johnston et al., 2020), species with weaker checklist spatial bias (i.e. higher scores) are likely better suited to a ‘ready‐to‐go’ approach, whereas species with stronger bias (i.e. lower scores) may require more nuanced modelling, or aggressive data filtering (e.g. Johnston et al., 2020).

### 2.3 Analysis

We analysed the above three data adequacy metrics across all 598 species. We analysed the two datasets (eBird and Birdata) both independently and combined. We explored (a) values of the three data adequacy metrics for the entire dataset; (b) cumulative (year‐on‐year) changes in the values of each of the three metrics for the period 1980–2022 (i.e. how these metrics change when more data are added to the two datasets each year) and (c) annual (year‐by‐year) changes in each of the three metrics across the same period.

### 2.4 Modelling

To further explore the factors associated with data adequacy, we developed a set of linear models to identify traits that may predict various adequacy metrics (Figure 1, panel e). We identified seven possible traits (Table 1) and constructed a simple linear model fit to each of the three adequacy metrics as follows (*N* = 3 models):

$$\begin{array}{cc} \text{Adequacy}\;= & \beta_{0}+\beta_{1}\times \text{threat status}+\beta_{2}\times \text{taxonomic uniqueness}\\ & +\beta_{3}\times \text{body mass}+\beta_{4}\times \text{average count}+\beta_{5}\times \text{range size}\\ & +\beta_{6}\times \text{species density}+\beta_{7}\times \text{human density}+\varepsilon \end{array}$$

where $\beta_{0}$ is the intercept of data adequacy (one of the three metrics defined above, for the entire combined dataset), $\beta_{1}$ is the partial regression slope of data adequacy on the species' International Union for Conservation of Nature (IUCN) threat status, as measured by the Action Plan for Australian Birds 2020 (Garnett & Baker, 2021), $\beta_{2}$ is the partial regression slope of data adequacy on the species' taxonomic uniqueness (a measure of how evolutionarily distinct a species is), as defined by Szabo et al. (2012) and presented in Garnett et al. (2015), $\beta_{3}$ is the partial regression slope of data adequacy on the species' average body mass, as presented in Garnett et al. (2015), $\beta_{4}$ is the partial regression slope of data adequacy on the average count of the species, calculated directly from the combined observation dataset used in the analysis, $\beta_{5}$ is the partial regression slope of data adequacy on the species' range size, calculated directly from the combined range maps used in the analysis, $\beta_{6}$ is the partial regression slope of data adequacy on the species' modelled density, as presented in Santini et al. (2023), $\beta_{7}$ is the partial regression slope of data adequacy on the average human population density (people/km^2^) across the species' range, calculated using data from Australian Bureau of Statistics (2023) and ε is the residual variation in data adequacy for individual species in the dataset.

**TABLE 1:** The seven traits used in modelling of the three data adequacy metrics.

Trait	Definition	Prediction	Reference
Threat status	IUCN Red List category (IUCN, 2001). Further details in Garnett and Baker (2021)	Higher threat status is associated with poorer data adequacy across all metrics. Threatened species typically have smaller population sizes (Mace et al., 2008), making them harder to find across their range, particularly for threatened species with remote ranges	Action plan for Australian birds 2020 (Garnett & Baker, 2021)
Taxonomic uniqueness	A constructed measure of taxon uniqueness. Further details in Garnett et al. (2015)	Higher taxonomic uniqueness is associated with better data adequacy across all metrics. Taxonomically unique species may be favoured by birdwatchers (Steven et al., 2017), resulting in distinctive species being disproportionately sought out and detected across their ranges	Defined by Szabo et al. (2012) and presented in Garnett et al. (2015)
Average body mass	Mean body mass (in grams) of all birds measured. Further details in Garnett et al. (2015)	Higher average body mass is associated with better data adequacy across all metrics. Larger birds are over‐represented in unstructured citizen science data (Callaghan, Poore, et al., 2021); this may also manifest in semi‐structured datasets as better data adequacy	Garnett et al. (2015)
Average count	Mean count (where reported) of observations of the species in the combined dataset	Higher average count is associated with better data adequacy across all metrics. Species that tend to occur in high numbers should be more readily detected across their ranges, resulting in better data adequacy scores	Calculated directly from the combined observation dataset
Range size	Area of combined, gridded and clipped distributions of the species in the dataset	Larger range size is associated with poorer data adequacy across all metrics. Larger ranges are inherently more difficult to comprehensively survey (Nandintsetseg et al., 2019) and are more likely to have high spatial sampling variation across the range	Calculated directly from the combined distribution dataset
Species density	Modelled species density across its whole range. Further details in Santini et al. (2023)	Higher species density is associated with better data adequacy across all metrics. Less scarce (higher density) species should be more readily detected across their ranges, resulting in better data adequacy scores	Santini et al. (2023)
Human density	Mean human density across the species' range	Higher human density is associated with better data adequacy across all metrics. Citizen science survey effort is strongly associated with population density (e.g. Botts et al., 2011); higher survey effort should yield more comprehensive coverage and more even effort	Calculated using data from Australian Bureau of Statistics (2023)

*Note*: A definition, reference and predicted relationship between trait and data adequacy is provided for each.

The six continuous predictor variables were all log‐transformed to satisfy assumptions of normality and linearity. Model assumptions were checked using the package performance (Lüdecke et al., 2021) and all modelling and analysis were conducted in R 4.2.0 (R Core Team, 2022).

## 3 RESULTS

### 3.1 Analysis

#### 3.1.1 Mean inventory completeness

**FIGURE 2:**
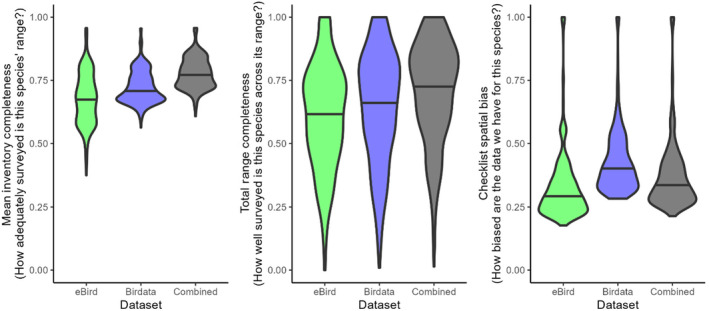
Violin plots of the distributions of the three data adequacy metrics calculated across the entire dataset (598 species) for the eBird (green), Birdata (blue) and combined (black) datasets.

**FIGURE 3:**
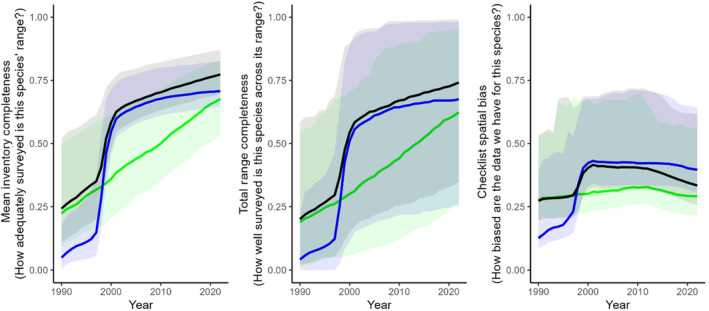
Cumulative (year‐on‐year) change of the three data adequacy metrics calculated for the eBird (green), Birdata (blue) and combined (black) datasets between 1990 and 2002. Solid lines are the median value across all species in each year; shaded ribbons denote the 5th–95th percentile range of values for all 598 species.

Mean inventory completeness values ranged between 0.61 and 0.96 for the entire combined dataset (mean 0.78, median 0.77; Table 2, Figure 2). The species with the lowest MIC values were all scarce, desert‐dwelling species with fairly large ranges (e.g. Dusky Grasswren *Amytornis purnelli*, Princess Parrot *Polytelis alexandrae*, Grey Honeyeater *Conopophila whitei*), whereas those with the highest scores were all range‐restricted island endemics (the highest‐scoring non‐island species was the Wet Tropics endemic Chowchilla *Orthonyx spaldingii* at 0.93). MIC values tended to be higher in the Birdata dataset than the eBird dataset (Figure 2), although at present growth rates this is likely to change by *c*. 2025 (Figure 3).

**TABLE 2:** Top and bottom 10 scores for each of the three data adequacy metrics across the combined cumulative dataset.

Mean inventory completeness			Total range completeness			Checklist spatial bias		
1	Cyanoramphus novaezelandiae Red‐crowned Parakeet	0.957	1	Gerygone modesta Norfolk Gerygone	1.000	1	Ducula whartoni Christmas Imperial‐Pigeon	1.000
2	*Gerygone modesta* Norfolk Gerygone	0.957	2	*Amaurornis phoenicurus* White‐breasted Waterhen	1.000	2	*Collocalia esculenta* Glossy Swiflet	1.000
3	*Ninox novaeseelandiae* Morepork	0.957	3	*Zosterops natalis* Christmas White‐eye	1.000	3	*Chalcophaps indica* Asian Emerald Dove	1.000
4	*Zosterops tenuirostris* Slender‐billed White‐eye	0.957	4	*Chalcophaps indica* Asian Emerald Dove	1.000	4	*Ducula mullerii* Collared Imperial‐Pigeon	1.000
5	*Amaurornis phoenicurus* White‐breasted Waterhen	0.957	5	*Collocalia esculenta* Glossy Swiftlet	1.000	5	*Ninox natalis* Christmas Boobook	1.000
6	*Zosterops natalis* Christmas White‐eye	0.957	6	*Ducula whartoni* Christmas Imperial‐Pigeon	1.000	6	*Aplonis cantoroides* Singing Starling	1.000
7	*Chionis minor* Black‐faced Sheathbill	0.955	7	*Ninox natalis* Christmas Boobook	1.000	7	*Dicaeum geelvinkianum* Red‐capped Flowerpecker	1.000
8	*Chalcophaps indica* Asian Emerald Dove	0.952	8	*Bolemoreus hindwoodi* Eungella Honeyeater	1.000	8	*Hypotaenidia sylvestris* Lord Howe Woodhen	1.000
9	*Collocalia esculenta* Glossy Swiftlet	0.952	9	*Hypotaenidia sylvestris* Lord Howe Woodhen	1.000	9	*Bolemoreus hindwoodi* Eungella Honeyeater	0.989
10	*Ducula whartoni* ^+1 other sp.^ Christmas Imperial‐Pigeon ^+1 other sp.^	0.952	10	*Aplonis cantoroides* ^+3 other spp.^ Singing Starling ^+3 other spp.^	1.000	10	*Gerygone modesta* ^+4 other spp.^ Norfolk Gerygone ^+4 other spp.^	0.987
…			…			…		
589	*Chlamydera guttata* Western Bowerbird	0.665	589	*Tyto novaehollandiae* Australian Masked Owl	0.203	589	*Radjah* Radjah Shelduck	0.250
590	*Poodytes carteri* Spinifexbird	0.654	590	*Hirundo rustica* Barn Swallow	0.199	590	*Cracticus nigrogularis* Pied Butcherbird	0.250
591	*Aphelocephala nigricincta* Banded Whiteface	0.651	591	*Cyclopsitta coxeni* Coxen's Fig‐Parrot	0.198	591	*Nettapus pulchellus* Green Pygmy Goose	0.249
592	*Amytornis striatus* Striated Grasswren	0.647	592	*Epthianura crocea* Yellow Chat	0.169	592	*Charadrius veredus* Oriental Plover	0.247
593	*Psophodes occidentalis* Western Whipbird	0.646	593	*Elanus scriptus* Letter‐winged Kite	0.132	593	*Numenius minutus* Little Curlew	0.246
594	*Neophema splendida* Scarlet‐chested Parrot	0.644	594	*Gallinago megala* Swinhoe's Snipe	0.132	594	*Centropus phasianinus* Pheasant Coucal	0.243
595	*Stipiturus ruficeps* Rufous‐crowned Emuwren	0.640	595	*Erythropitta macklotii* Papuan Pitta	0.111	595	*Chlamydera cerviniventris* Fawn‐breasted Bowerbird	0.238
596	*Conopophila whitei* Grey Honeyeater	0.635	596	*Polytelis alexandrae* Princess Parrot	0.096	596	*Trichodere cockerelli* White‐streaked Honeyeater	0.234
597	*Polytelis alexandrae* Princess Parrot	0.608	597	*Turnix olivii* Buff‐breasted Buttonquail	0.069	597	*Grus antigone* Sarus Crane	0.228
598	*Amytornis purnelli* Dusky Grasswren	0.608	598	*Pezoporus occidentalis* Night Parrot	0.014	598	*Cracticus mentalis* Black‐backed Butcherbird	0.214

*Note*: Full version is available in Appendix S2.

**FIGURE 4:**
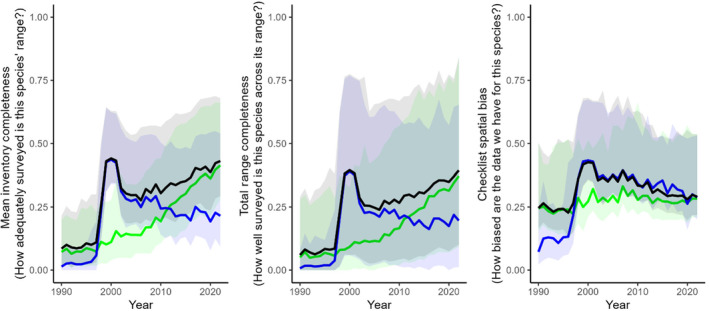
Annual (year‐by‐year) change of the three data adequacy metrics calculated for the eBird (green), Birdata (blue) and combined (black) datasets between 1990 and 2002. Solid lines are the median value across all species in each year; shaded ribbons denote the 5th 95th percentile range of values for all 598 species.

Annual MIC values for the combined dataset ranged between an average of 0.10 in 1990 and 0.46 in 2000 (Figure 4). Prior to 1998 and after 2011, the eBird dataset had higher average MIC values, whereas between these years the Birdata dataset dominated, particularly between 1999 and 2001.

#### 3.1.2 Total range completeness

Total range completeness values ranged between 0.01 and 1.00 for the entire combined dataset (mean 0.71, median 0.74; Table 2, Figure 2). The species with the lowest TRC values were all either very rare, very cryptic or both (e.g. Night Parrot *Pezoporus occidentalis*, Buff‐breasted Buttonquail *Turnix olivii*, Princess Parrot *Polytelis alexandrae*), whereas those with the highest scores were primarily island endemics (two range‐restricted mainland species, Eungella Honeyeater *Bolemoreus hindwoodi* and Yellow‐spotted Honeyeater *Meliphaga notata*, also had TRC values of 1). TRC values tended to be higher in the Birdata dataset than in the eBird dataset, although this is likely to change by *c*. 2025 with present growth rates (Figure 3).

Annual TRC values for the combined dataset ranged between an average of 0.09 in 1990 and 0.42 in 2022 (Figure 4). Prior to 1998 and after 2011, the eBird dataset had higher average TRC values, whereas between these years the Birdata dataset dominated, particularly between 1999 and 2001.

#### 3.1.3 Checklist spatial bias

Checklist spatial bias values ranged between 0.21 and 1.00 for the combined dataset (mean 0.37, median 0.33; Table 2, Figure 2). The species with the lowest CSB values (i.e. strongest spatial bias) shared few obvious traits, but were often wide‐ranging species with distributions that straddled inland Australia, where survey effort is generally lower, and the densely populated east coastal fringe, where survey effort is generally higher (e.g. Pheasant Coucal *Centropus phasianinus*, Pied Butcherbird *Cracticus nigrogularis*, Chestnut‐breasted Mannikin *Lonchura castaneothorax*). Species with the highest scores (i.e. weakest spatial bias) were all island endemics (the highest‐scoring non‐island species was Eungella Honeyeater *Bolemorus hindwoodi* at 0.99). Spatial bias tended to be stronger (lower CSB score) in the eBird dataset for most species (Figures 2 and 3).

Annual CSB values for the combined dataset ranged between an average of 0.25 in 1993 and 0.46 in 2000 (Figure 4). Prior to 1998, spatial bias was typically worse (lower score) in the Birdata dataset; since then, it has been typically worse in the eBird dataset with the exception of 2020, when both datasets had approximately equal spatial bias (*c*. 0.30).

### 3.2 Modelling

We tested the relationship between seven different species traits and the three measures of adequacy, fitting models for 561 species. Thirty‐seven species had missing values that could not be imputed for at least one of the seven predictor variables and therefore were not included. The majority of missing values (35/37 species) came from the density dataset (Santini et al., 2023), with the remaining missing values coming from the body mass dataset (Garnett et al., 2015). For the most part, these 37 species did not share many common traits, although several poorly known species (e.g. Buff‐breasted Buttonquail *T. olivii*, Coxen's Fig‐Parrot *Cyclopsitta coxeni*) were included in this group; a full list of all 37 species with missing values is provided in Appendix S3.

**FIGURE 5:**
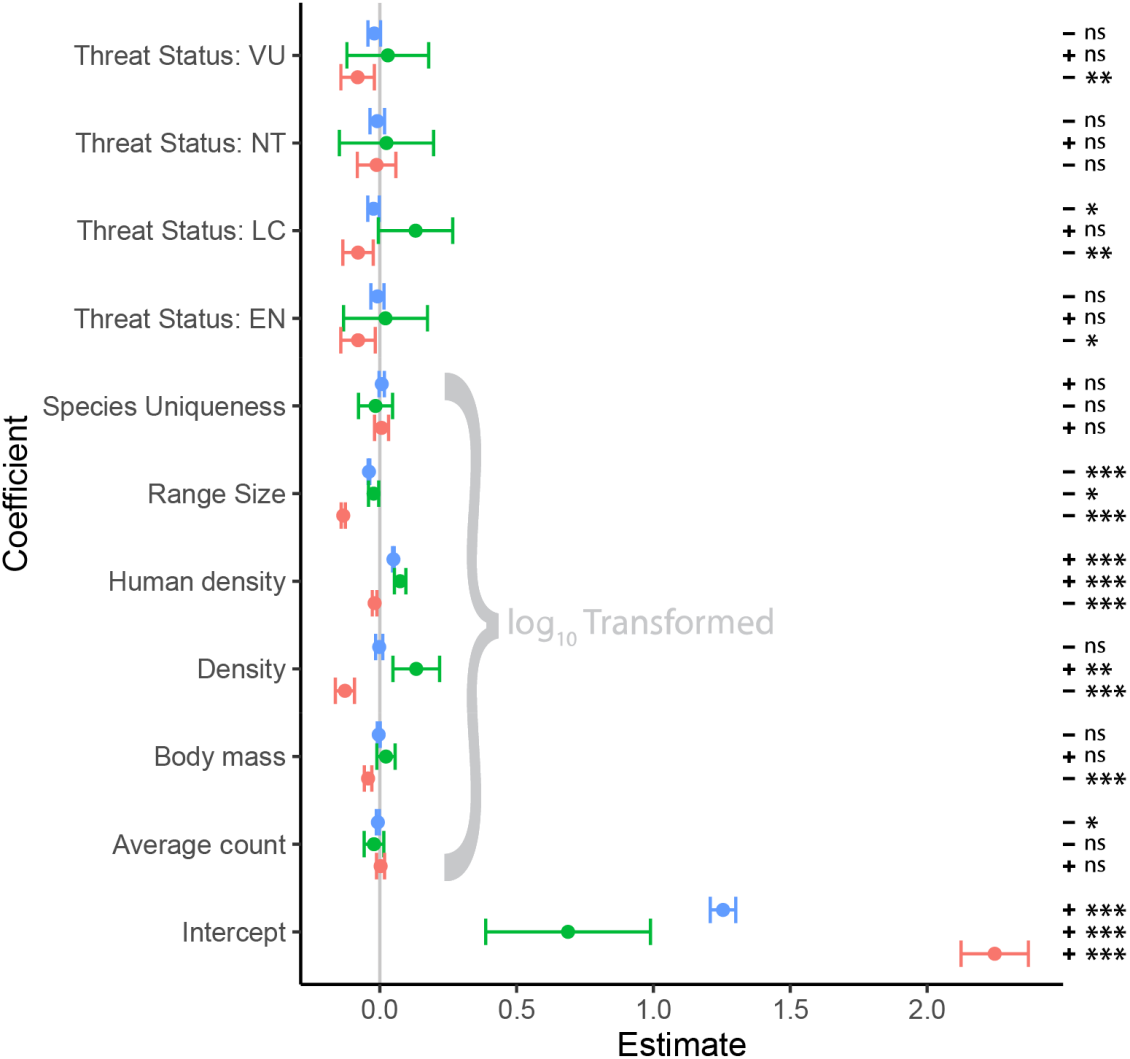
Coefficient estimates (effect sizes) and 95% confidence intervals for the three models (one per data adequacy metric). Blue = Mean Inventory Completeness; Green = Total Range Completeness; Red = Checklist Spatial Bias. A negative coefficient means decreased data adequacy (as measured by the specific metric) as the value of the predictor variable increases, or in comparison to the reference state of the predictor variable (CR for Threat Status). NB: the six continuous predictor variables were all log‐transformed to satisfy assumptions of normality and linearity. *p*‐value cut‐offs: *p* < .001: ***; .001 ≤ *p* < .01: **; .01 ≤ *p* < .05: *; *p* ≥ .05: ns.

The fitted models mostly had good explanatory power, with *R* ^2^ values ranging from .14 for total range completeness (adjusted *R* ^2^ = .14, *F*_10,550_ = 9.902, *p* = 2.5 × 10^−15^) to .72 for checklist spatial bias (adjusted *R* ^2^ = .72, *F*_10,550_ = 142.9, *p* < 2.2 × 10^−16^) and .79 for mean inventory completeness (adjusted *R* ^2^ = .79, *F*_10,550_ = 211.2, *p* < 2.2 × 10^−16^). Effect sizes for most coefficients tended to be fairly small, and about half (16/30) were non‐significant (Figure 5). Across the adequacy metrics, range size was a statistically significant predictor. Species with larger range sizes tended to have lower mean inventory completeness (*p* < 2.2 × 10^−16^), total range completeness (*p* < 2.2 × 10^−16^) and checklist spatial bias (*p* < 2.2 × 10^−16^). Similarly, average human population density was a statistically significant predictor across all three metrics, with species with more densely populated ranges typically having higher mean inventory completeness (*p* < 2.2 × 10^−16^) and total range completeness (*p* < 2.2 × 10^−16^), but lower checklist spatial bias (*p* = 1.9 × 10^−7^). Conversely, species' taxonomic uniqueness was not a statistically significant predictor for any metric. The relationships between the other coefficients and the three metrics were generally more mixed. Complete results for all model coefficients are presented in Figure 5.

## 4 DISCUSSION

We used three metrics of adequacy to explore the monitoring capacity of citizen science data from two major Australian programmes, eBird and Birdata. Data adequacy—as we have defined it—is a broadly applicable and repeatable way of assessing the potential utility of a dataset prior to conducting any subsequent analyses, allowing analysts and end users to identify and prioritise species and regions that will benefit most from further monitoring efforts. We found that mean inventory completeness and total range completeness are both higher in the Birdata dataset than in the eBird dataset for most species, despite the significantly greater volume of data in the eBird dataset. This paradoxical result is likely driven by the stronger spatial bias (lower checklist spatial bias scores) in the eBird dataset, which has relatively lower survey effort across much of remote Australia, but much higher effort in more densely populated regions. As a consequence of this, citizen science data coverage for many Australian bird species is still incomplete, especially those whose ranges span the arid zone. However, mean inventory completeness and total range completeness are both steadily increasing year‐by‐year for most species, primarily due to the continuing rapid expansion of eBird use by the birdwatching community in the country.

Two of our metrics (mean inventory completeness and total range completeness) are alike in that, cumulatively (year‐on‐year), they can only increase (and have mostly also increased year‐by‐year, as noted above). However, the third metric we present (checklist spatial bias) is not constrained in the same way and has worsened across both datasets in recent years (Figures 3 and 4). This has implications for analysts wishing to use citizen science data, as spatial bias needs to be considered and controlled for in any analyses since uncontrolled spatial biases can lead to unreliable or incorrect model inference (Backstrom, 2022; Johnston et al., 2020). Fortunately, the effects of increased spatial bias (i.e. worsening checklist spatial bias) may be mitigated by the exponential growth in data volume (i.e. increasing mean inventory completeness and total range completeness), as larger data volumes across species' ranges allow analysts to use filtering and subsampling methods that account for these worsening biases while retaining a sufficiently large sample size (see e.g. Johnston et al., 2021). Furthermore, spatial bias in the eBird dataset may be stabilising, likely reflective of consistent observer practices over time, with growth now driven mainly by new users rather than increased survey effort per user. Such insights provide valuable direction for analysts wishing to use these datasets and are enhanced by the synergy achieved by exploring multiple metrics simultaneously.

The temporal context provided in our analysis also highlights the impact that guided approaches to citizen science can have on data adequacy and overall dataset quality (Callaghan, Poore, et al., 2019; Callaghan, Rowley, et al., 2019; Callaghan, Watson, et al., 2021). This is best demonstrated by the considerable increase in all three adequacy metrics in the Birdata dataset during the period 1999–2001 (Figure 4), corresponding with the New Atlas of Australian Birds project run by Birdlife Australia (Barrett et al., 2003). During this period, survey effort in remote Australia was considerably greater than any period since, resulting in meaningfully increased data adequacy scores across all three metrics. Similar guided approaches to target poorly represented species or regions could be implemented in either eBird or Birdata in the future, with their effectiveness assessed simply by repeating the analyses presented here. Indeed, since our approach is not limited to any region or taxonomic group, it may be used to broadly monitor the state of knowledge for any group for which there have been citizen science efforts, targeted or otherwise.

Our methods thus allow for species to be classified according to how suitable available citizen science data currently are, for any given application. For some species, currently available citizen science data may be ‘ready to go’, allowing for populations of these species to be modelled and tracked without significant further in‐field investment, especially if other data sources (e.g. pre‐existing structured surveys) are integrated into models. However, for many other species, particularly those that are rare or cryptic, or those with large or remote ranges, current citizen science data are insufficient, and in many cases, broad‐scale citizen science projects like eBird and Birdata are unlikely to ever be sufficient unless more targeted approaches are employed. The potential of such targeted approaches is readily demonstrated in our analyses by the considerable increase in all three metrics in the Birdata dataset during the period corresponding with the New Atlas of Australian Birds project. Finally, for especially data‐poor species where targeted citizen science approaches are either unavailable or unsuccessful, more formal monitoring methods will likely be necessary to fill in the gaps.

In addition to providing values for each of the metrics we present, we also explored the relationship between each of the three metrics and several species‐specific traits (Table 1, Figure 5). We found various patterns in the relationships between species' traits and the three adequacy metrics we assess. These include the unsurprising finding that a species' range size has strong implications for how well‐sampled a species is, indicating that species with large range sizes are inherently more difficult to fully sample and quantify than those with small range sizes, supporting other research (Nandintsetseg et al., 2019). Similarly, we found a positive relationship between human population density and two of our adequacy metrics (mean inventory completeness and total range completeness), further supporting the body of literature that has found a strong bias in citizen science data towards human settlements (e.g. Botts et al., 2011). This indicates that more ‘remote’ species are most likely to need prioritisation in biodiversity research and targeted monitoring efforts. Surprisingly, taxonomic uniqueness was not strongly correlated with any of our three metrics.

Although we are satisfied with the robustness of our analyses, we do note a small number of limitations. One issue is the spatial grain size used. The sizes of terrestrial Australian bird distributions span more than five orders of magnitude, ranging from highly range‐restricted island endemics to species found across the entire continent; even among mainland taxa, the range is more than two orders of magnitude (the Eungella Honeyeater having the smallest range, being found across just *c*. 0.26% of the continent). In some instances, small spatial grain may be important, particularly if the desired downstream analyses or management applications require a similarly small grain. However, the overall trends we observe (increasing mean inventory completeness and total range completeness, but worsening checklist Spatial Bias) are consistent across a range of meaningful grain sizes (0.2–2.0 degrees; Figures S1–S4). As such, while the absolute values of the three metrics tend to decrease with increasingly fine‐scale grain sizes, this is not an issue as the relative relationships remain intact.

A second limitation to note is that while we present three metrics of data adequacy, these are not necessarily a complete inventory of all possible ways to quantify adequacy (we do not, e.g. attempt to explicitly quantify adequacy in the temporal context), nor are the three presented necessarily essential. Depending on the intended research question(s), some metrics may be more useful than others. The commonality between all data adequacy metrics, however, is their ability to provide additional context when exploring a biodiversity dataset, and in particular their ability to identify gaps—parts of the dataset with poor adequacy—that may need to be addressed in any downstream applications, for example, by integrating citizen science data with other sources.

By developing and presenting methods to quantify the adequacy of citizen science datasets for biodiversity monitoring, we show it is possible to identify species‐specific biases and gaps. Conservation efforts are typically most successful when done at the species level (Ward et al., 2020), and so taxonomically precise assessments of data adequacy are important in deriving appropriate conclusions from observational datasets that can be directly applied to conservation outcomes. Species‐specific measures of data adequacy—a key area of novelty in our analysis—allow for species to be classified or prioritised according to an analyst's specific needs and are especially critical in any conservation‐minded assessment of citizen science data.

## AUTHOR CONTRIBUTIONS

**Louis J. Backstrom:** Conceptualization (lead); formal analysis (lead); methodology (lead); project administration (equal); visualization (lead); writing – original draft (lead). **Corey T. Callaghan:** Conceptualization (supporting); methodology (supporting); writing – original draft (supporting). **Nicholas P. Leseberg:** Conceptualization (supporting); methodology (supporting); writing – original draft (supporting). **Chris Sanderson:** Conceptualization (supporting); writing – review and editing (equal). **Richard A. Fuller:** Conceptualization (supporting); supervision (supporting); writing – review and editing (equal). **James E. M. Watson:** Conceptualization (supporting); methodology (supporting); project administration (equal); supervision (lead); writing – original draft (supporting).

## CONFLICT OF INTEREST STATEMENT

The authors declare that they have no known competing financial interests or personal relationships that could have appeared to influence the work reported in this paper.

## Supporting information


Appendix S1.

Appendix S2.

Appendix S3.

## Data Availability

All datasets used have been cited in the text. A GitHub repository with full code and outputs is available at https://github.com/Louis‐Backstrom/DataAdequacy. Key R packages used include auk (Strimas‐Mackey et al., 2021), tidyverse (Wickham et al., 2019) and sf (Pebesma, 2018).
