# Supplementary material for: Assessing adequacy of citizen science datasets for biodiversity monitoring
Source: Ecol Evol. 2024 Jan 31;14(2):e10857. doi: 10.1002/ece3.10857 (PMC10830347; doi:10.1002/ece3.10857)
Supplement: Supplementary file 1 — Appendix S1. [file ECE3-14-e10857-s003.docx]

# Appendix

|  | **MIC** | **TRC** | **CSB** |
| --- | --- | --- | --- |
| **2.0** | 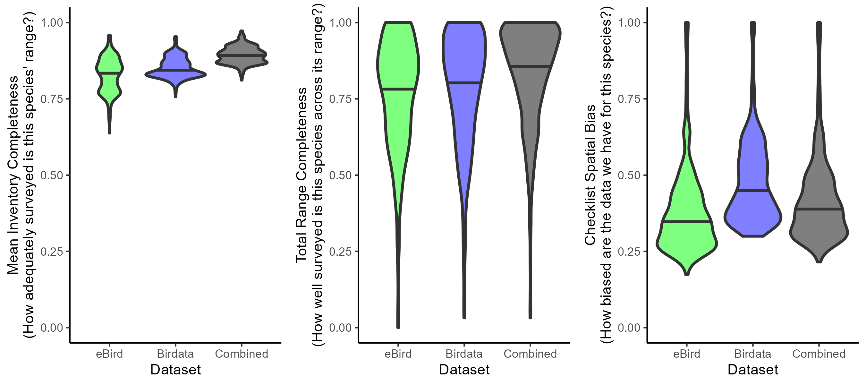 | | |
| **1.0** | 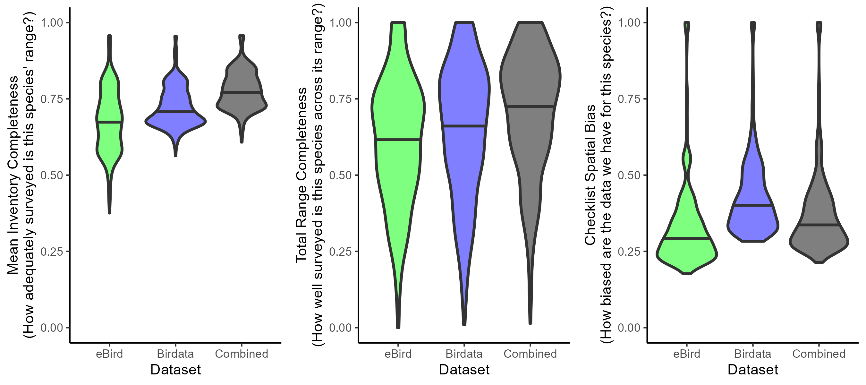 | | |
| **0.5** | 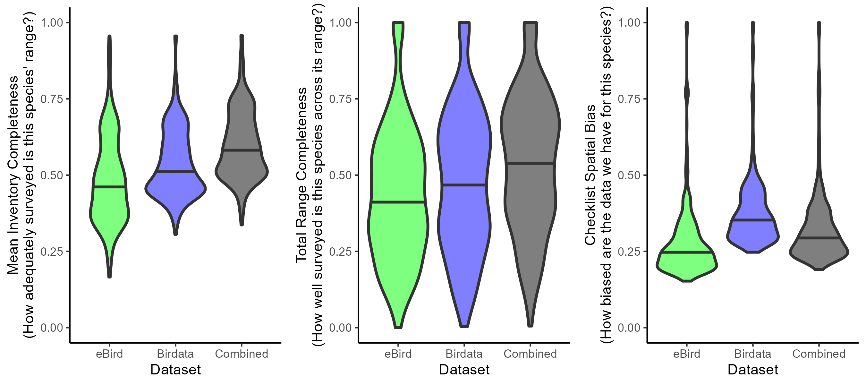 | | |
| **0.2** | 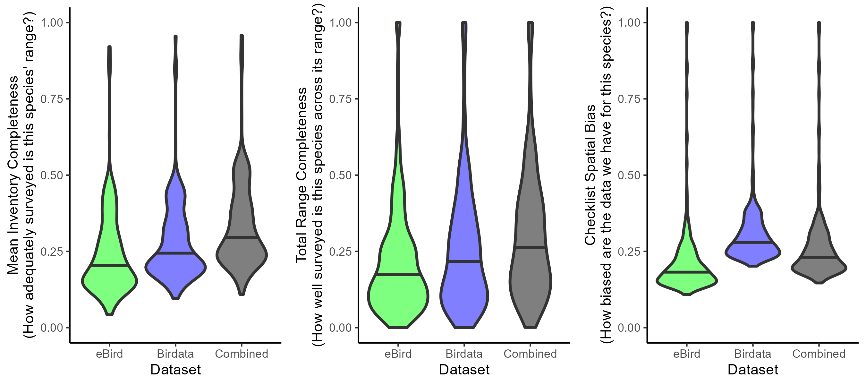 | | |

*Appendix Figure 1 Comparison of violin plots (see Figure 2) across four spatial grain sizes: 2.0 degrees, 1.0 degrees, 0.5 degrees, and 0.2 degrees.*

|  | **MIC** | **TRC** | **CSB** |
| --- | --- | --- | --- |
| **2.0** | 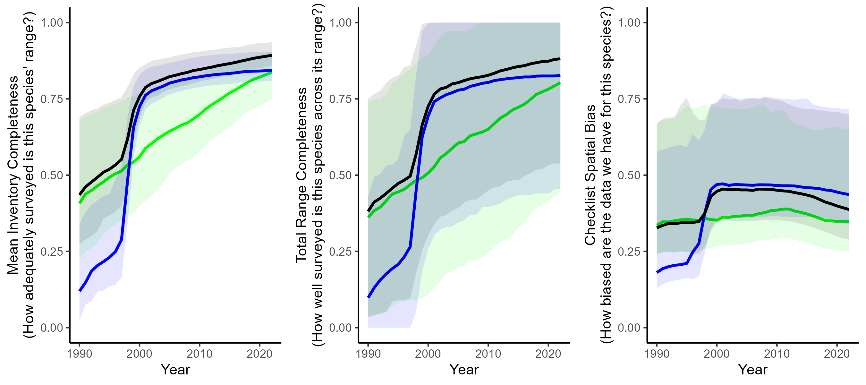 | | |
| **1.0** | 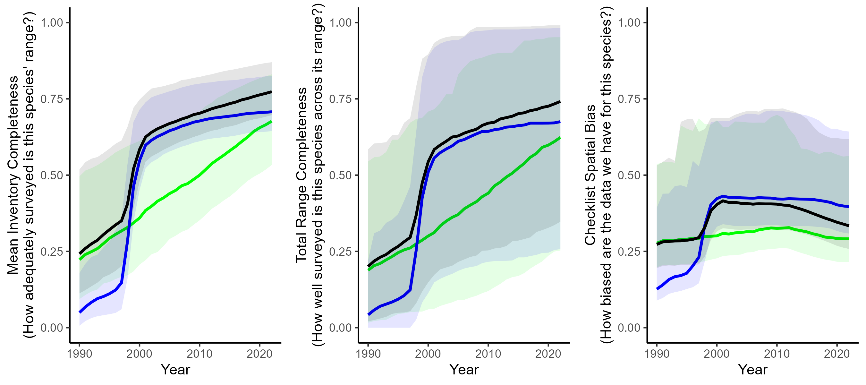 | | |
| **0.5** | 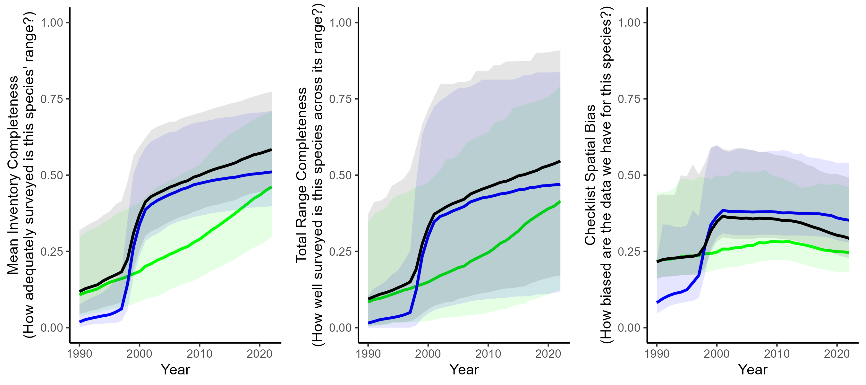 | | |
| **0.2** | 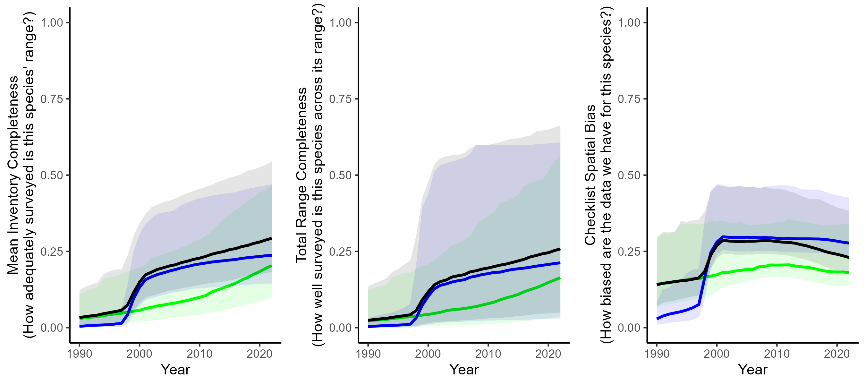 | | |

*Appendix Figure 2 Comparison of cumulative (year-on-year) change plots (see Figure 3) across four spatial grain sizes: 2.0 degrees, 1.0 degrees, 0.5 degrees, and 0.2 degrees.*

|  | **MIC** | **TRC** | **CSB** |
| --- | --- | --- | --- |
| **2.0** | 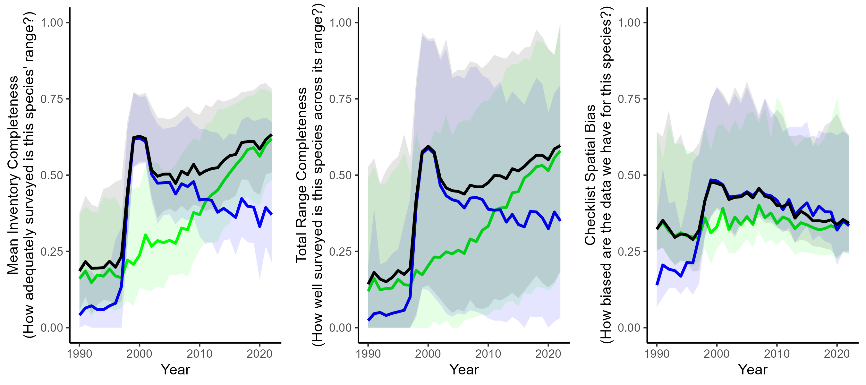 | | |
| **1.0** | 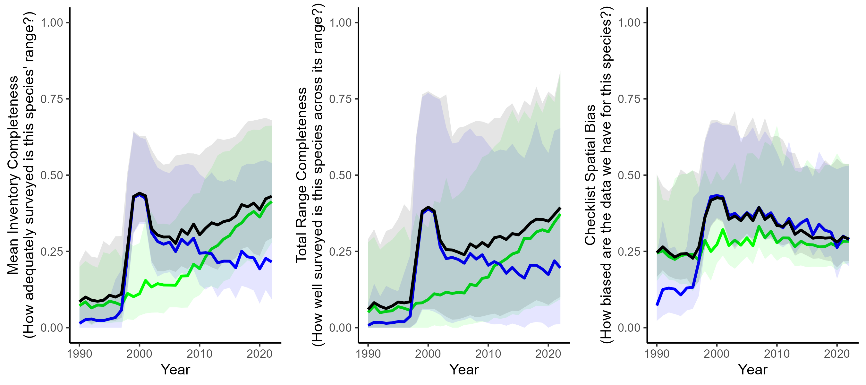 | | |
| **0.5** | 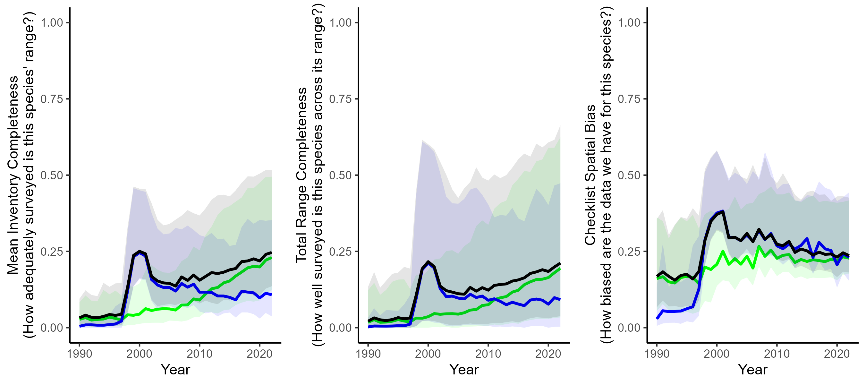 | | |
| **0.2** | 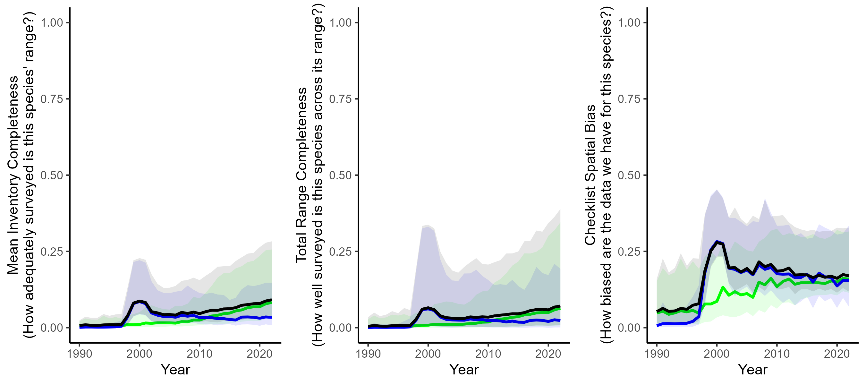 | | |

*Appendix Figure 3 Comparison of annual (year-by-year) change plots (see Figure 4) across four spatial grain sizes: 2.0 degrees, 1.0 degrees, 0.5 degrees, and 0.2 degrees.*

| **2.0** | **1.0** | **0.5** | **0.2** |
| --- | --- | --- | --- |
| **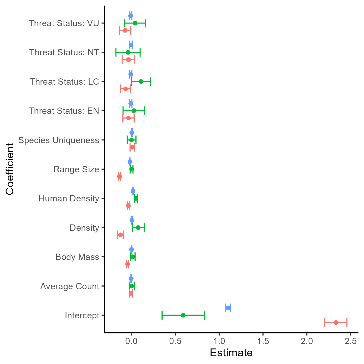** | **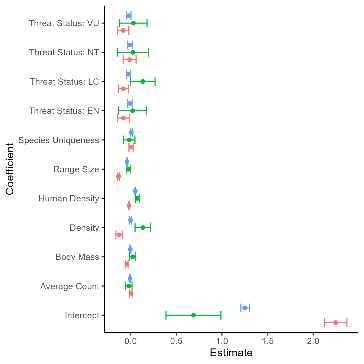** | **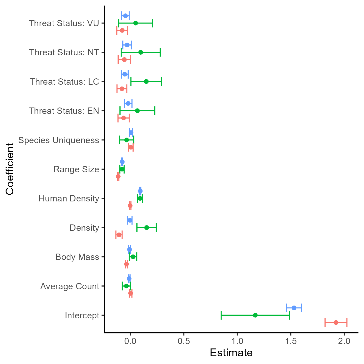** | **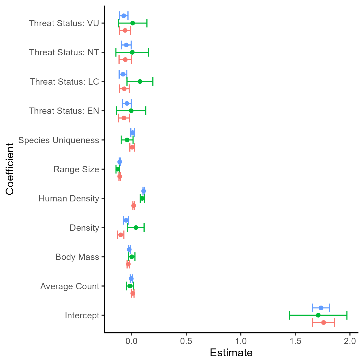** |

*Appendix Figure 4 Comparison of coefficient estimates (see Figure 5) across four spatial grain sizes: 2.0 degrees, 1.0 degrees, 0.5 degrees, and 0.2 degrees.*
